# Data on German farmers risk preference, perception and management strategies

**DOI:** 10.1016/j.dib.2017.09.014

**Published:** 2017-09-14

**Authors:** Manuela Meraner, Robert Finger

**Affiliations:** a Agricultural Economics and Policy Group, ETH Zurich, Zurich, Switzerland; b Production Economics Group, Institute for Food and Resource Economics, University of Bonn, Bonn, Germany

## Abstract

The extent to which people are willing to take on risk, i.e. their risk preferences as well as subjective risk perception plays a major role in explaining their behavior. This is of particular relevance in agricultural production, which is inherently risky. The data presented here was collected amongst a total of 64 German farmers in 2015. It includes results of three different risk preference elicitation methods (multiple price list, business statements in four relevant domains and general self-assessment) as well as risk perception. Additionally, farm business characteristics (e.g. size, farm-level workforce, succession) and personal farmer characteristics (e.g. age, gender, risk literacy) are included.

**Specifications Table**

**Table:** 

Subject area	Experimental Economics, Agricultural Risk Management
More specific subject area	*Risk preference elicitation, risk perception*
Type of data	*CSV File*
How data was acquired	*Paper-pencil survey*
Data format	*Raw data, partially analyzed*
Experimental factors	*No pretreatment of sample*
Experimental features	*Very brief experimental description*
Data source location	*North-Rhine Westphalia, Germany*
Data accessibility	*With this article*

**Value of the data**

• The data allows for comparison of risk preferences with other case studies in meta-analyses.
• The data allows for comparison of risk perception of agricultural risks amongst farmers in a meta-analysis.
• The data allows for linking risk preferences, risk perception and risk literacy to applied risk management strategies.

• The data allows for comparison of risk literacy measured using subjective numeracy.

## 1 Data

The data includes results from a paper pencil survey sample of 64 German livestock farmers from North-Rhine Westphalia. We include the farmers subjective risk perception of 25 risk sources. Additionally, the data includes three different risk preference elicitation methods: a contextualized multiple price list (MPL) following Holt and Laury [1], four business statements measuring relative risk preferences in four different domains following Meuwissen, Huirne, and Hardaker [2] and a general self-assessment of risk preferences following Dohmen et al. [3]. Furthermore, it includes information on the farmers' subjective numeracy and ability to process probabilistic information. Moreover, it includes farmers experience with risk and their applied risk management strategies as well as information on their business, personal and household characteristics. The data was first analyzed in the article by Meraner and Finger [4].

## 2 Experimental design, materials and methods

A paper-pencil survey was distributed by extension service employees in two rounds, with the first at the beginning of December 2015 and the second at the beginning of January 2016. The return cutoff date was 31. December and 31. January respectively in the North and North-East of North-Rhine Westphalia. In the survey a stamped, self-addressed envelope is included, leading to a total of 64 responses (representing a 26% response rate). In total 11 (6%) of returns where partial in the sense that at least one question was not answered, no returns were excluded. The survey consisted of 31 questions structured along the following six topical sections:

i) subjective perception of risk
ii) risk preference elicitation
iii) farmer's characteristics
iv) household characteristics
v) information about the farm business
vi) risk management tools used

Participating farmers could indicate to get a feedback regarding their risk perceptions, attitudes and management strategies, as well as aggregated information on the whole sample.

Subjective risk perception is measured via 25 Likert type questions on the perceived likelihood of a risk source on a scale from 1 to 5 where 1=very unlikely and 5=very likely and, 25 questions on the perceived potential negative impact in the case of its occurrence on a scale from 1 to 5 where 1=very small impact and 5=very strong impact. The perceived risk scores can be calculated by multiplying the perceived probability of occurrence with the perceived impact [2], [5], [6]. The choice of included risk domains is based on the in-depth expert interviews with two extension service consultants and two farmers as well as a literature study [2], [6], [7].

**Fig. 1:**
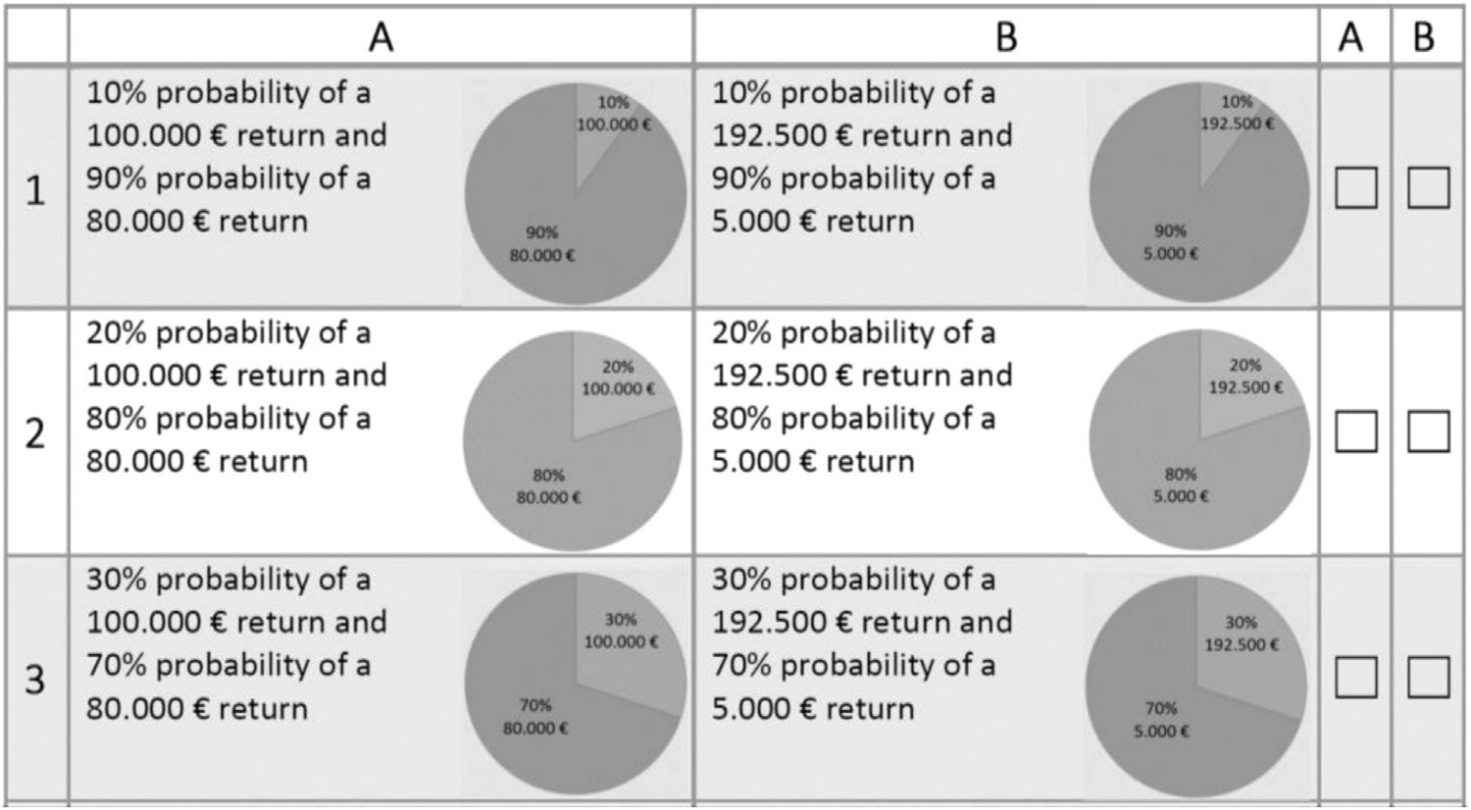
Example visual presentation MPL.

Risk preferences are elicited using three methods that are dominantly used in the literature. First, a contextualized multiple price list (MPL) was used following Holt and Laury [1]. Here the original lottery wording is changed to an agricultural decision wording. The presented question introducing the MPL reads as following: “Assume that you are offered to make an agricultural investment. Here you will get with different associated probabilities for investment A a return of 100,000 € or 80,000 € and for investment B a return of 192,500 € or 5000 €. You can choose in the following table in each row between the two investment options (A or B).” Participants were instructed that real payouts are reconverted by the factor of one thousand. We create a realistic payout structure of the contextualized MPL and divide the lottery prices by 1.000 so that real payouts range from 5€ to 192.5€.The upscaling of payouts does not change the original CRRA intervals used by Holt and Laury [1] but creates an incentive compatible MPL. The expected return for each participating farmer is 9.50€, the average time to complete the questionnaire was estimated at 20 min, resulting in an hourly wage equivalent of 28.50€. For the payout procedure we chose between-subjects random incentive system following [8], [9] and informed farmers that at the end of the survey period 10% of all participants are selected for real payouts based on their choices. The randomly chosen 10% of participants were informed via the channel they indicated on the survey (e-mail or post) about their prize and payed out via bank transfer. Furthermore we reduce complexity and consequential inconsistent behavior by including a pie chart displaying proportions next to the verbal presentation of decisions as a visual aid [10], [11] (an example of the visual presentation is found in Fig. 1).

Secondly, we elicit farmers relative risk preferences in four central risk domains in agriculture (i.e. four business statements). Following [2], [5], [6], [12] the Likert based questions ask about the farmers' agreement with the following four statements: “I am willing to take more risks than my colleagues with respect to … i) production risks, … ii) marketing and pricing risks, … iii) financial risks and … iv) farming in general on a scale from 1 to 5 where 1 means “fully agree” and 5 means “don't agree”.

Thirdly, a Likert scale question on the farmer's general self-assessment of risk preferences on a scale from 0 to 10 where 0 means “not at all willing to take risks” and 10 means “very willing to take risks” following [3] is included. In contrast to the MPL, the risk preference elicitation methods based on the business statements and self-assessment are not financially incentivized.

We elicit risk literacy by using a set of seven Likert type questions on numerical aptitude and preferences for numbers, adapted from Fagerlin et al. [13] to measure the farmers' subjective numeracy and ability to process probabilistic information. German translations are taken from Garcia-Retamero and Galesic [14].

The survey also included questions on the farmers' age, gender, years of farming experience, level of education, participation in agricultural training sessions, training on risk management specifically and experienced past losses in five different domains (i.e. marketing and prices, production, finances, labor and public acceptance). Additionally, to capture the general level of optimism we included two questions addressing the farmers' current life satisfaction and their predicted life satisfaction in one year. On the household level, we included information on the farms’ total work force availability, farm succession and household size. The collected farm business information includes the type of agricultural production (organic/conventional), occupancy of the farmer (full-time/part-time), the farm size (agricultural area), usage of the area, the proportion of rented land, and size and type of livestock, postal code. Moreover, it includes a list of 17 applied risk management strategies.

The full questionnaire in German and English as well as Variable names and descriptions are included in the Supplementary file.
