# Supplementary material for: Data on German farmers risk preference, perception and management strategies
Source: Data Brief. 2017 Sep 14;15:102–5. doi: 10.1016/j.dib.2017.09.014 (PMC5614755; doi:10.1016/j.dib.2017.09.014)
Supplement: Supplementary file 4 — Supplementary material [file mmc4.docx]

| **Frage** | **Question** | **Variable name** | **scaling** |
| --- | --- | --- | --- |
|  |  | Nr. | ID, number 1-64 |
| 1. Risikofaktoren | Risk factors |  |  |
| - 1. Wie schätzen Sie die Eintrittswahrscheinlichkeit der folgenden Risikoquellen für Ihren Betrieb ein? | - 1. **How would you assess the probability of the following sources of risk for your business? (From 1 = "very unlikely" to 5 = "very likely")** |  |  |
|  |  | Mean likelihood market price risks | scale from 1 to 5  1 = very unlikely  5 = very likely |
| Zunehmende Preisschwankungen auf den Absatzmärkten | **Increasing price volatility on the outlet markets** | 1.1Price volatility outlet markets | scale from 1 to 5  1 = very unlikely  5 = very likely |
| Zunehmende Preisschwankungen auf den Beschaffungsmärkten | **Increasing price volatility on the purchasing markets** | 1.2Price volatility purchasing markets | scale from 1 to 5  1 = very unlikely  5 = very likely |
| Steigende Pachtpreise für Land | **Increasing farm land prices** | 1.3Rental prices | scale from 1 to 5  1 = very unlikely  5 = very likely |
| Steigende Futtermittelpreise | **Increasing feed prices** | 1.4Feed prices | scale from 1 to 5  1 = very unlikely  5 = very likely |
| Schwächung der Erzeuger durch hohe Marktmacht der Abnehmer | **Weakening of the producers due to high market power of buyers** | 1.5Market power of single actors | scale from 1 to 5  1 = very unlikely  5 = very likely |
|  |  | Mean likelihood political structural risks | scale from 1 to 5  1 = very unlikely  5 = very likely |
| Weitere Senkungen der EU Direktzahlungen | **Further reductions of EU direct payments** | 1.6Direct payments | scale from 1 to 5  1 = very unlikely  5 = very likely |
| Verschärfung von Cross Compliance | **Intensification of cross compliance** | 1.7Cross compliance | scale from 1 to 5  1 = very unlikely  5 = very likely |
| Steigende Auflagen in der Tierproduktion(Tierschutz) | **Increasing requirements in animal production (animal welfare law)** | 1.8Animal welfare | scale from 1 to 5  1 = very unlikely  5 = very likely |
| Steigende Auflagen in der Pflanzenproduktion (Umweltschutz) | **Increasing requirements in plant production (environmental protection)** | 1.9Nature conservation | scale from 1 to 5  1 = very unlikely  5 = very likely |
| Weiterer Abbau der EU-Marktstützung(z. Bsp. Außenschutz, Intervention, usw.) | **Further reduction of the EU market support (e.g. border protection, intervention, etc.)** | 1.10EU market protection | scale from 1 to 5  1 = very unlikely  5 = very likely |
| Weitere Ökologisierung der Agrarpolitik | **Further greening of agricultural policy** | 1.11Ecologisation | scale from 1 to 5  1 = very unlikely  5 = very likely |
| Einschränkungen im landwirtschaftlichen Baurecht | **Limitations in agricultural construction law** | 1.12Construction law | scale from 1 to 5  1 = very unlikely  5 = very likely |
| Plötzlicher Wegfall von Absatz- und Bezugsmärkten | **Sudden loss of outlet and procurement markets** | 1.13Elimination of markets | scale from 1 to 5  1 = very unlikely  5 = very likely |
|  |  | Mean likelihood production risks | scale from 1 to 5  1 = very unlikely  5 = very likely |
| Ertragsschwankungen aufgrund von Klimaänderungen | **Yield fluctuations due to climate change** | 1.14Climate change | scale from 1 to 5  1 = very unlikely  5 = very likely |
| Ertragsausfälle aufgrund von klimatischen Extremereignissen(z. Bsp. Hochwasser, Hagel) | **Yield losses due to extreme climatic events (e.g. flood, hail)** | 1.15Extreme weather events | scale from 1 to 5  1 = very unlikely  5 = very likely |
| **Tierseuchen und Tierkrankheiten** | **Animal epidemics and animal diseases** | 1.16Animal diseases | scale from 1 to 5  1 = very unlikely  5 = very likely |
| Schwierigkeiten in der Bekämpfung von Schadorganismen (Resistenzen) | **Difficulties in controlling harmful organisms (resistance)** | 1.17Resistencies | scale from 1 to 5  1 = very unlikely  5 = very likely |
| Verringerte Flächenverfügbarkeit | **Reduced land availability** | 1.18Less land availability | scale from 1 to 5  1 = very unlikely  5 = very likely |
|  |  | Mean likelihood financial risks | scale from 1 to 5  1 = very unlikely  5 = very likely |
| Liquiditätsengpässe | **Liquidity shortages** | 1.19Liquidity shortfalls | scale from 1 to 5  1 = very unlikely  5 = very likely |
| Abnahme der Kreditwürdigkeit | **Decrease in creditworthiness** | 1.20Decreasing credit status | scale from 1 to 5  1 = very unlikely  5 = very likely |
|  |  | Mean likelihood workforce legal risks | scale from 1 to 5  1 = very unlikely  5 = very likely |
| Eingeschränkte Verfügbarkeit qualifizierter Arbeitskräfte | **Limited availability of skilled staff** | 1.21Finding workforce | scale from 1 to 5  1 = very unlikely  5 = very likely |
| Ausfall von Führungskräften im Betrieb | **Drop out of business leader** | 1.22Loss main farm operator | scale from 1 to 5  1 = very unlikely  5 = very likely |
| Probleme bei der Einhaltung von Qualitätsanforderungen | **Problems with compliance with quality requirements** | 1.23Quality control | scale from 1 to 5  1 = very unlikely  5 = very likely |
| Akzeptanzprobleme der Tierhaltung  (z.B. Widerstand gegen Stallneubauten) | **Acceptance problems of animal husbandry (e.g. resistance to building of new stables)** | 1.24Acceptance life stock | scale from 1 to 5  1 = very unlikely  5 = very likely |
| Akzeptanzprobleme des Ackerbaus  (z.B. Vermaisung der Landschaft) | **Acceptance problems of agriculture**  **(e.g. domination of maize in the landscape )** | 1.25Acceptance arable farming | scale from 1 to 5  1 = very unlikely  5 = very likely |
| - 1. Wie schätzen Sie die Schadensauswirkung der folgenden Risikoquellen für Ihren Betrieb ein (von 1 = „keine Auswirkungen“ bis 5 = „existenzgefährdend“)? | - 1. **How would you rate the potential of damage for the following sources of risk to your business? (From 1 = "very unlikely" to 5 = "very likely").** |  |  |
|  |  | Mean impact market price risks | scale from 1 to 5  1 = very small impact  5 = very strong impact |
| Zunehmende Preisschwankungen auf den Absatzmärkten | **Increasing price volatility on the outlet markets** | 2.1Price volatility outlet markets | scale from 1 to 5  1 = very small impact  5 = very strong impact |
| Zunehmende Preisschwankungen auf den Beschaffungsmärkten | **Increasing price volatility on the purchasing markets** | 2.2Price volatility purchasing markets | scale from 1 to 5  1 = very small impact  5 = very strong impact |
| Steigende Pachtpreise für Land | **Increasing farm land prices** | 2.3Rental prices | scale from 1 to 5  1 = very small impact  5 = very strong impact |
| Steigende Futtermittelpreise | **Increasing feed prices** | 2.4Feed prices | scale from 1 to 5  1 = very small impact  5 = very strong impact |
| Schwächung der Erzeuger durch hohe Marktmacht der Abnehmer | **Weakening of the producers due to high market power of buyers** | 2.5Market power of single actors | scale from 1 to 5  1 = very small impact  5 = very strong impact |
|  |  | Mean impact political structural risks | scale from 1 to 5  1 = very small impact  5 = very strong impact |
| Weitere Senkungen der EU Direktzahlungen | **Further reductions of the EU direct payments** | 2.6Direct payments | scale from 1 to 5  1 = very small impact  5 = very strong impact |
| Verschärfung von Cross Compliance | **Intensification of cross compliance** | 2.7Cross compliance | scale from 1 to 5  1 = very small impact  5 = very strong impact |
| Steigende Auflagen in der Tierproduktion(Tierschutz) | **Increasing requirements in animal production (animal welfare law)** | 2.8Animal welfare | scale from 1 to 5  1 = very small impact  5 = very strong impact |
| Steigende Auflagen in der Pflanzenproduktion (Umweltschutz) | **Increasing requirements in plant production (environmental protection)** | 2.9Nature conservation | scale from 1 to 5  1 = very small impact  5 = very strong impact |
| Weiterer Abbau der EU-Marktstützung(z. Bsp. Außenschutz, Intervention, usw.) | **Further reduction of the EU market support (e.g. border protection, intervention, etc.)** | 2.10EU market protection | scale from 1 to 5  1 = very small impact  5 = very strong impact |
| Weitere Ökologisierung der Agrarpolitik | **Further greening of agricultural policy** | 2.11Ecologisation | scale from 1 to 5  1 = very small impact  5 = very strong impact |
| Einschränkungen im landwirtschaftlichen Baurecht | **Limitations in agricultural construction law** | 2.12Construction law | scale from 1 to 5  1 = very small impact  5 = very strong impact |
| Plötzlicher Wegfall von Absatz- und Bezugsmärkten | **Sudden loss of outlet and procurement markets** | 2.13Elimination of markets | scale from 1 to 5  1 = very small impact  5 = very strong impact |
|  |  | Mean impact production risks | scale from 1 to 5  1 = very small impact  5 = very strong impact |
| Ertragsschwankungen aufgrund von Klimaänderungen | **Yield fluctuations due to climate change** | 2.14Climate change | scale from 1 to 5  1 = very small impact  5 = very strong impact |
| Ertragsausfälle aufgrund von klimatischen Extremereignissen(z. Bsp. Hochwasser, Hagel) | **Yield losses due to extreme climatic events (e.g. flood, hail)** | 2.15Extreme weather events | scale from 1 to 5  1 = very small impact  5 = very strong impact |
| Tierseuchen und Tierkrankheiten | **Animal epidemics and animal diseases** | 2.16Animal diseases | scale from 1 to 5  1 = very small impact  5 = very strong impact |
| Schwierigkeiten in der Bekämpfung von Schadorganismen (Resistenzen) | **Difficulties in controlling harmful organisms (resistance)** | 2.17Resistencies | scale from 1 to 5  1 = very small impact  5 = very strong impact |
| Verringerte Flächenverfügbarkeit | **Reduced land availability** | 2.18Less land availability | scale from 1 to 5  1 = very small impact  5 = very strong impact |
|  |  | Mean impact financial risks | scale from 1 to 5  1 = very small impact  5 = very strong impact |
| Liquiditätsengpässe | **Liquidity shortages** | 2.19Liquidity shortfalls | scale from 1 to 5  1 = very small impact  5 = very strong impact |
| Abnahme der Kreditwürdigkeit | **Decrease in creditworthiness** | 2.20Decreasing credit status | scale from 1 to 5  1 = very small impact  5 = very strong impact |
|  |  | Mean impact workforce legal risks | scale from 1 to 5  1 = very small impact  5 = very strong impact |
| Eingeschränkte Verfügbarkeit qualifizierter Arbeitskräfte | **Limited availability of skilled staff** | 2.21Finding workforce | scale from 1 to 5  1 = very small impact  5 = very strong impact |
| Ausfall von Führungskräften im Betrieb | **Drop out of business leader** | 2.22Loss main farm operator | scale from 1 to 5  1 = very small impact  5 = very strong impact |
| Probleme bei der Einhaltung von Qualitätsanforderungen | **Problems with compliance with quality requirements** | 2.23Quality control | scale from 1 to 5  1 = very small impact  5 = very strong impact |
| Akzeptanzprobleme der Tierhaltung  (z.B. Widerstand gegen Stallneubauten) | **Acceptance problems of animal husbandry (For example, resistance to building new stables)** | 2.24Acceptance life stock | scale from 1 to 5  1 = very small impact  5 = very strong impact |
| Akzeptanzprobleme des Ackerbaus  (z.B. Vermaisung der Landschaft) | **Acceptance problems of agriculture**  **(For example domination of maize in the landscape )** | 2.25Acceptance arable farming | scale from 1 to 5  1 = very small impact  5 = very strong impact |
|  |  | Perceived Risk 1 | 1.1*2.1 |
|  |  | Perceived Risk 2 | 1.2*2.2 |
|  |  | Perceived Risk 3 | 1.3*2.3 |
|  |  | Perceived Risk 4 | 1.4*2.4 |
|  |  | Perceived Risk 5 | 1.5*2.5 |
|  |  | Perceived mp risk | average perceived market and price risks 1-5 |
|  |  | Perceived Risk 6 | 1.6*2.6 |
|  |  | Perceived Risk 7 | 1.7*2.7 |
|  |  | Perceived Risk 8 | 1.8*2.8 |
|  |  | Perceived Risk 9 | 1.9*2.9 |
|  |  | Perceived Risk 10 | 1.10*2.10 |
|  |  | Perceived Risk 11 | 1.11*2.11 |
|  |  | Perceived Risk 12 | 1.12*2.12 |
|  |  | Perceived Risk 13 | 1.13*2.13 |
|  |  | Perceived pol risk | average perceived political / structural risks 6-13 |
|  |  | Perceived Risk 14 | 1.14*2.14 |
|  |  | Perceived Risk 15 | 1.15*2.15 |
|  |  | Perceived Risk 16 | 1.16*2.16 |
|  |  | Perceived Risk 17 | 1.17*2.17 |
|  |  | Perceived Risk 18 | 1.18*2.18 |
|  |  | Perceived prod risk | average perceived production risks 14-18 |
|  |  | Perceived Risk 19 | 1.19*2.19 |
|  |  | Perceived Risk 20 | 1.20*2.20 |
|  |  | Perceived fin risk | average perceived financial risks 19-20 |
|  |  | Perceived Risk 21 | 1.21*2.21 |
|  |  | Perceived Risk 22 | 1.22*2.22 |
|  |  | Perceived Risk 23 | 1.23*2.23 |
|  |  | Perceived Risk 24 | 1.24*2.24 |
|  |  | Perceived Risk 25 | 1.25*2.25 |
|  |  | Perceived son risk | average perceived workforce legal risks 21-25 |
| 1. Risikoeinstellung | Attitude towards risk |  |  |
| - 1. Nehmen Sie an, Ihnen wird angeboten eine landwirtschaftliche Investition zu tätigen. Dabei erhalten Sie mit bestimmten Wahrscheinlichkeiten für Investition A eine Auszahlung von 100.000 € oder 80.000 € und für Investition B eine Auszahlung von 192.500 € oder 5.000 €. Beide Investitionen unterscheiden sich nicht bezüglich der Kosten und Auszahlungszeitpunkte. Sie können in der folgenden Tabelle in jeder Zeile zwischen den zwei Investitionsentscheidungen (A oder B) wählen. | - 1. **Assume that you are offered to make an agricultural investment. Your return is associated with different probabilities. For investment A a return of 100.000 € or 80.000 € and for investment B a return of 192.500 € or 5.000 €. In the following table choose between the two investment-options (A or B) in each row.** |  |  |
| - - 1. A: 10% Wahrscheinlichkeit für eine Auszahlung von 100.000 € und 90% Wahrscheinlichkeit für eine Auszahlung von 80.000 €   B: 10% Wahrscheinlichkeit für eine Auszahlung von 192.500 € und 90% Wahrscheinlichkeit für eine Auszahlung von 5.000 € | **B.1.1. A: 10% probability of a 100.000 € return and 90% probability of a 80.000 € return**  **B: 10% probability of a 192.500 € return and 90% probability of a 5.000 € return** | HL1 | 1 if A  0 if B |
| - - 1. A: 20% Wahrscheinlichkeit für eine Auszahlung von 100.000 € und 80% Wahrscheinlichkeit für eine Auszahlung von 80.000 €   B: 20% Wahrscheinlichkeit für eine Auszahlung von 192.500 € und 80% Wahrscheinlichkeit für eine Auszahlung von 5.000 € | **B.1.2. A: 20% probability of a 100.000 € return and 80% probability of a 80.000 € return**  **B: 20% probability of a 192.500 € return and 80% probability of a 5.000 € return** | HL2 | 1 if A  0 if B |
| - - 1. A: 30% Wahrscheinlichkeit für eine Auszahlung von 100.000 € und 70% Wahrscheinlichkeit für eine Auszahlung von 80.000 €   B: 30% Wahrscheinlichkeit für eine Auszahlung von 192.500 € und 70% Wahrscheinlichkeit für eine Auszahlung von 5.000 € | **B.1.3. A: 30% probability of a 100.000 € return and 70% probability of a 80.000 € return**  **B: 30% probability of a 192.500 € return and 70% probability of a 5.000 € return** | HL3 | 1 if A  0 if B |
| - - 1. A: 40% Wahrscheinlichkeit für eine Auszahlung von 100.000 € und 60% Wahrscheinlichkeit für eine Auszahlung von 80.000 €   B: 40% Wahrscheinlichkeit für eine Auszahlung von 192.500 € und 60% Wahrscheinlichkeit für eine Auszahlung von 5.000 € | **B.1.4. A: 40% probability of a 100.000 € return and 60% probability of a 80.000 € return**  **B: 40% probability of a 192.500 € return and 60% probability of a 5.000 € return** | HL4 | 1 if A  0 if B |
| - - 1. A: 50% Wahrscheinlichkeit für eine Auszahlung von 100.000 € und 50% Wahrscheinlichkeit für eine Auszahlung von 80.000 €   B: 50% Wahrscheinlichkeit für eine Auszahlung von 192.500 € und 50% Wahrscheinlichkeit für eine Auszahlung von 5.000 € | **B.1.5. A: 50% probability of a 100.000 € return and 50% probability of a 80.000 € return**  **B: 50% probability of a 192.500 € return and 50% probability of a 5.000 € return** | HL5 | 1 if A  0 if B |
| - - 1. A: 60% Wahrscheinlichkeit für eine Auszahlung von 100.000 € und 40% Wahrscheinlichkeit für eine Auszahlung von 80.000 €   B: 60% Wahrscheinlichkeit für eine Auszahlung von 192.500 € und 40% Wahrscheinlichkeit für eine Auszahlung von 5.000 € | **B.1.6. A: 60% probability of a 100.000 € return and 40% probability of a 80.000 € return**  **B: 60% probability of a 192.500 € return and 40% probability of a 5.000 € return** | HL6 | 1 if A  0 if B |
| - - 1. A: 70% Wahrscheinlichkeit für eine Auszahlung von 100.000 € und 30% Wahrscheinlichkeit für eine Auszahlung von 80.000 €   B: 70% Wahrscheinlichkeit für eine Auszahlung von 192.500 € und 30% Wahrscheinlichkeit für eine Auszahlung von 5.000 € | **B.1.7. A: 70% probability of a 100.000 € return and 30% probability of a 80.000 € return**  **B: 70% probability of a 192.500 € return and 30% probability of a 5.000 € return** | HL7 | 1 if A  0 if B |
| - - 1. A: 80% Wahrscheinlichkeit für eine Auszahlung von 100.000 € und 20% Wahrscheinlichkeit für eine Auszahlung von 80.000 €   B: 80% Wahrscheinlichkeit für eine Auszahlung von 192.500 € und 20% Wahrscheinlichkeit für eine Auszahlung von 5.000 € | **B.1.8. A: 80% probability of a 100.000 € return and 20% probability of a 80.000 € return**  **B: 80% probability of a 192.500 € return and 20% probability of a 5.000 € return** | HL8 | 1 if A  0 if B |
| - - 1. A: 90% Wahrscheinlichkeit für eine Auszahlung von 100.000 € und 10% Wahrscheinlichkeit für eine Auszahlung von 80.000 €   B: 90% Wahrscheinlichkeit für eine Auszahlung von 192.500 € und 10% Wahrscheinlichkeit für eine Auszahlung von 5.000 € | **B.1.9. A: 90% probability of a 100.000 € return and 10% probability of a 80.000 € return**  **B: 90% probability of a 192.500 € return and 10% probability of a 5.000 € return** | HL9 | 1 if A  0 if B |
| - - 1. A: 100% Wahrscheinlichkeit für eine Auszahlung von 100.000 € und 0% Wahrscheinlichkeit für eine Auszahlung von 80.000 €   B: 100% Wahrscheinlichkeit für eine Auszahlung von 192.500 € und 0% Wahrscheinlichkeit für eine Auszahlung von 5.000 € | **B.1.10. A: 100% probability of a 100.000 € return and 0% probability of a 80.000 € return**  **B: 100% probability of a 192.500 € return and 0% probability of a 5.000 € return** | HL10 | 1 if A  0 if B |
|  |  | Lottery A count | sum of A in mpl |
|  |  | CRRA interval mid point count | CRRA interval mid point resulting from A count^[[1]](#footnote-1)^ |
|  |  | Lottery switching point | row of first switch from A to B in mpl |
|  |  | CRRA interval mid point switch | crra interval mid point resulting from first switching row^[[2]](#footnote-2)^ |
| - 1. Wie schätzen Sie sich persönlich ein: Sind Sie im Allgemeinen ein risikobereiter Mensch oder versuchen Sie, Risiken zu vermeiden? (von 0 = „gar nicht risikobereit“ bis 10 = „sehr risikobereit“). | B.2. How do you see yourself personally: Are you generally a risk-averse person, or do you try to avoid risks?  **(from 0 = "risk averse" to 10 = "very risk loving").** | SA | scale from 0 to 10  0 = “very risk averse”  10 = “very risk loving” |
| - 1. Wie zufrieden sind Sie gegenwärtig, alles in allem, mit Ihrem Leben? (von 1 = „unzufrieden“ bis 10 = „zufrieden“) | B.3. Please rate your general current life satisfaction (from 1 = "very unhappy" to 10 = "very happy") | Life sat | scale from 1 to 10  1 = “very unhappy”  10 = “very happy” |
| - 1. Und was glauben Sie, wie wird es wohl in einem Jahr sein? (von 1 = „unzufrieden“ bis 10 = „zufrieden“) | B.4. How do you predict this to be in a year from now? (from 1 = "very unhappy" to 10 = "very happy") | Life sat future | scale from 1 to 10  1 = “very unhappy”  10 = “very happy” |
|  |  | opt | life sat future - life sat |
| - 1. Bitte geben Sie in der folgenden Tabelle an inwiefern Sie den Aussagen zustimmen (von 1 = „stimme voll zu“ bis 5 = „lehne ab“). | B.5. Plase match your opinion to the statements in the table below. (from 1 = strongly agree to 5 = strongly disagree) |  |  |
| - - 1. Ich bin bereit, in Bezug auf die Produktion mehr Risiken einzugehen als andere Landwirte. | B.5.1. Compared to other farmers, I am willing to take more risk in terms of production. | BS 1 | scale from 1 to 5  1 = fully agree  5 = disagree |
| - - 1. Ich bin bereit, in Bezug auf Markt- und Preise mehr Risiken einzugehen als andere Landwirte. | B.5.2. Compared to other farmers, I am willing to take more risk in terms of marketing and prices. | BS 2 | scale from 1 to 5  1 = fully agree  5 = disagree |
| - - 1. Ich bin bereit, in Bezug auf Fremdkapitalaufnahme mehr Risiken einzugehen als andere Landwirte. | B.5.3. Compared to other farmers, I am willing to take more risk in terms of financial risks | BS 3 | scale from 1 to 5  1 = fully agree  5 = disagree |
| - - 1. Ich bin bereit, in Bezug auf Landwirtschaft generell mehr Risiken einzugehen als andere Landwirte. | B.5.4. Compared to other farmers, I am willing to take more risk in agriculture generally | BS 4 | scale from 1 to 5  1 = fully agree  5 = disagree |
|  |  | BS average |  |
| - 1. Durch welchen der folgenden Faktoren hatten Sie in den letzten 5 Jahren größere Verluste auf Ihrem landwirtschaftlichen Betrieb (mehrfach Nennung möglich)? | B.6. For the past 5 years, which of the following factors was caused major losses on your farm? |  |  |
| - 1. Markt- und Preisrisiken | B.7. Market and price risks | exp MP risk | 1 if yes |
| - 1. Politikänderungen | B.8. Political and structural risks | exp pol risk | 1 if yes |
| - 1. Produktionsrisiken | B.9. Production risks | exp prod risk | 1 if yes |
| - 1. Finanzielle Risiken | B.10. Financial risks | exp fin risk | 1 if yes |
| - 1. Risiken durch Arbeitskräfte, gesellschaftliche Akzeptanz | B.11. Risks associated with workforce or public acceptance | exp workforce risk | 1 if yes |
| - 1. keine größeren Verluste in den letzten 5 Jahren | B.12. No major losses over the last 5 years | exp none | 1 if yes |
| - 1. Größere Verluste in den letzten 5 Jahren | B.13.. Experienced any major losses over the last five years | exp losses | 1 if yes |
| 1. Umgang mit Wahrscheinlichkeiten | C. Handling probabilities |  |  |
| - 1. Wie schätzen Sie sich im Bruchrechnen ein? | C.1. How good are you at working with fractions? | SNT1 | scale from 1 to 6  1 = extremely good  6 = not good at all |
| - 1. Wie schätzen Sie Ihre Fähigkeiten im Umgang mit Prozentwerten ein? | C.2. How good are you at working with percentages? | SNT2 | scale from 1 to 6  1 = extremely good  6 = not good at all |
| - 1. Wie gut sind Sie darin einzuschätzen, wie viel ein Ferkel bei einer 25%-gen Preisreduzierung kostet? | C.3. How good are you at figuring out how much a piglet will cost if it prices dropped by 25%? | SNT3 | scale from 1 to 6  1 = extremely good  6 = not good at all |
| - 1. Wenn Sie eine Tageszeitung lesen, wie nützlich finden Sie Tabellen und Diagramme als Teil eines Artikels? | C.4. When reading the newspaper, how helpful do you find tables and graphs that are parts of a story? | SNT4 | scale from 1 to 6  1 = extremely helpful  6 = not at all helpful |
| - 1. Wenn Ihnen jemand etwas über die Wahrscheinlichkeit erzählt, dass ein bestimmtes Ereignis eintreffen wird, bevorzugen Sie es dann, wenn dazu Worte benutzt werden („passiert selten“) oder wenn Zahlenwerte benutzt werden („es gibt eine 1%ige Wahrscheinlichkeit“) | C.5. When people tell you the probability of an event, do you prefer that they use words (‘‘it rarely happens’’) or numbers (‘‘there’s a 1% probability’’)? | SNT5 | scale from 1 to 6  1 = always prefer words  6 = always prefer numbers |
| - 1. Wenn Sie einen Wetterbericht hören, bevorzugen Sie es dann, wenn die Vorhersagen in Pro-zentwerten ausgedrückt werden (z.B. „es gibt heute eine 20%ige Regenwahrscheinlichkeit“) oder in Worten( z.B. „heute ist die Regenwahrscheinlichkeit gering“)? | C.6. When you hear a weather forecast, do you prefer predictions using percentages (e.g., ‘‘there will be a 20% probability of rain today’’) or predictions using only words (e.g., ‘‘there is a small probability of rain today’’)? | SNT6 | scale from 1 to 6  1 = always prefer words  6 = always prefer percentages |
| - 1. Wie oft finden Sie Informationen, die in Zahlen ausgedrückt sind, nützlich? | C.7. How often do you find numerical information to be useful? | SNT7 | scale from 1 to 6  1 = never  6 = very often |
|  |  | Mean SN | higher values mean less risk literacy |
| 1. Betriebliche Informationen | D. Operational information |  |  |
| - 1. Wird Ihr landwirtschaftlicher Betrieb im Haupt- oder im Nebenerwerb geführt? | D.1. Are you working your farm as a full-, or part-time farmer? | Full time | 1 if full time farmer |
| - 1. Wird Ihr landwirtschaftlicher Betrieb konventionell, oder ökologisch bewirtschaftet? | D.2. Is your farm organic or conventional? | organic | 1 if organic farm |
| - 1. Landw. genutzte Fläche | D.3. Agricultural land | agric area | ha of agricultural area |
| - - 1. davon Eigentum | D.3.1. of which owned land | ownership | ha of owned land |
| - - 1. davon Pacht | D.3.2. of which rented land | rented land | ownership of agricultural area |
|  |  | rent ratio | agricultural area/rented land |
| - 1. Ackerfläche | D.4. Arable land | arab | ha of arable land |
| - - 1. davon Sonderkulturen | D.4.1. of which horticulture | hort | ha of horticulture |
| - 1. Grünland | D.5. Greenland | greenland | ha of greenland |
| - 1. Welchen Tierbestand gibt es in Ihrem Betrieb? | D.6. What’s the amount of livestock on your farm? |  |  |
| - - 1. Zuchtsauen | D.6.1. Breeding sows | breeding sows | nr of breeding sows |
| - - 1. Mastschweine (ab 25kg) | D.6.2. Fattening pigs | fattening pigs | nr of fattening pigs |
| - - 1. Ferkel | D.6.3. Piglets | piglets | nr of piglets |
| - - 1. Milchvieh | D.6.4. Dairy cows | dairy cows | nr of dairy cows |
| - - 1. Mastbullen | D.6.5. Fattening bull | fattening bull | nr of fattening bulls |
| - - 1. Weibliche Rinder | D.6.6. Female cattle | female cattle | nr of female cattle |
| - - 1. Mastgeflügel | D.6.7. Fattening poultry | fattening poultry | nr of fattening poultry |
| - - 1. Legehennen | D.6.8. Laying hens | laying hens | nr of laying hens |
| - - 1. Pferde | D.6.9. Horses | horses | nr of horses |
| - - 1. Schweine | D.6.10. Pigs | pig | sum of breeding sows, fattening pigs, piglets |
| - - 1. Rinder | D.6.11 Cattle | cattle | sum of dairy cows, fattening bull, female cattle |
| - 1. Ist auf Ihrem Betrieb die Hofnachfolge gesichert? | D.7. Is the succesion for your farm secured? |  |  |
| - - 1. Nachfolge sicher | D.7.1. Succession sure | SU1 | 1 if succession is planned and sure |
| - - 1. Nachfolge ziemlich sicher | D.7.2. Succession quite sure | SU2 | 1 if succession is planned and quite sure |
| - - 1. Nachfolge ziemlich unsicher | D.7.3. Succession quite unsure | SU3 | 1 if succession is planned and quite unsure |
| - - 1. Nachfolge unsicher | D.7.4. Succession unsure | SU4 | 1 if succession is planned, but unsure |
| - - 1. Keine Nachfolge geplant | D.7.5. Succession not planned | SU5 | 1 if succession is not planned in the next 15 years |
| - - 1. Nachfolge nicht erforderlich / Betriebsaufgabe | D.7.6. Succession not required / stop farming | SU6 | 1 if succession is not required |
|  |  | SU | 1 if succession is planned and sure  0. 5 if succession is planned and quite sure  -0. 5 if succession is planned and quite unsure  -1 if succession is planned, but unsure  0 if succession is not planned in the next 15 years |
| 1. Risikomanagementstrategien | E. Risk management strategies |  |  |
| - 1. Risikoangepasste Produktionsweise (z. B. Vorsichtige Wahl der Aussaatzeitpunkte) | E.1. Risk adapted production (e.g. careful selection of sowing dates) | prod | 1 if applied |
| - 1. Nutzung resistenter Sorten oder robuster Rassen | E.2. Use of robust or resistant races or types | resis | 1 if applied |
| - 1. Landwirtschaftliche Diversifizierung (z. B. Kombinationen von Winter und Sommergetreiden, Tierhaltung und Pflanzenanbau) | E.3. Agricultural diversification (e.g. combination of summer and winter grains, livestock and agriculture) | agric div | 1 if applied |
| - 1. Landwirtschaftsnahe Diversifizierung (z. B. Direktvermarktung, Fremdenverkehr, Hof Café, Energieerzeugung, Sonstiges) | E.4. Non-agricultural diversification (e.g. direct marketing, agritourism, gastronomy, renewable energies, others) | nonagric div | 1 if applied |
| - 1. Investitionen in Technologien, die meine Produktionsbedingungen besser an das Wetter anpassen (z. B. Bewässerung) | E.5. Investment in new technologies for improved weather adaptation (e.g. irrigation | inv | 1 if applied |
| - 1. Bildung von Liquiditätsreserven | E.6. Building reserves | liq | 1 if applied |
| - 1. Härter Arbeiten /private Ausgaben kürzen | E.7. Work harder or cut private expenses | work harder | 1 if applied |
| - 1. Kooperationen mit anderen Betrieben | E.8. Cooperation with other farmers | coop | 1 if applied |
| - 1. Arbeiten außerhalb des Betriebs | E.9. Off-farm work | off farm work | 1 if applied |
| - 1. Investitionen außerhalb des Betriebs (z.B. andere Unternehmungen, Immobilien, Wertanlagen) | E.10. Off-farm investment | off farm inv | 1 if applied |
| - 1. Betriebshaftpflichtversicherung | E.11. Public liability insurance | pl ins | 1 if applied |
| - 1. Ertragsschadenversicherung (z.B. Tierversicherung) | E.12. Yield insurance | yield ins | 1 if applied |
| - 1. Hagelversicherung | E.13. Hail insurance | hail ins | 1 if applied |
| - 1. Mehrgefahrenversicherung (z.B. Hagel + Sturm und Starkregen, Hagel + Sturm und Starkregen + Starkfrost) | E.14. Multiple risk insurance | multiple ins | 1 if applied |
| - 1. Betriebshaftpflichtversicherung + Bodenkasko, erweiterte Umwelthaftpflicht | E.15. Environmental risks insurance | environment ins | 1 if applied |
| - 1. Rechtschutzversicherung | E.16. Legal expense insurance | legal ins | 1 if applied |
| - 1. Warenterminbörsen | E.17. Trading on the commodity futures exchange | comm | 1 if applied |
| - 1. Keine dieser Maßnahmen | E.18. No risk management tool | no RM | 1 if applied |
|  |  | sum RM tools | sum of all risk management tools applied |
|  |  | agnonagoff | 0 if mean off-farm strategies max  1 if mean on farm agriculture max  2 if on farm non agriculture max |
| 1. Betriebsleitung, Familie und Arbeitskräfte | F. Management, Family and workers |  |  |
| - 1. Wie viele Personen einschließlich Ihnen leben in Ihrem Haushalt (inkl. Altenteiler)? | F.1. How many people, including yourself, are living in the farm household? | hhize | number of people living in the farm household |
| - 1. In welchem Jahr sind Sie geboren? | F.2. What is your year of birth? | year birth | year of birth |
|  |  | age | age in 2016 |
| - 1. Geschlecht | F.3. Gender of the farmer | gender | 1 = male  0 = female |
| - 1. Wie viele Arbeitskräfte arbeiten insgesamt Vollzeit auf Ihrem landwirtschaftlichen Betrieb (inkl. Familienarbeitskräfte)? | F.4. What is the number of people working in your business? (including family workers) | workforce | workforce working on the farm |
| - 1. In welchem Jahr haben Sie den Betrieb übernommen? | F.5. In what year did you take over the business? | experience | year of business take over |
| - 1. Bitte geben Sie Ihre Postleitzahl an: | F.6. What is the postal code of the farms location? | CAP | postal code of farm location |
| - 1. Bitte geben Sie Ihren höchsten Bildungsabschluss an. | F.7. What is your highest educational degree? | educ | highest educational degree in German schooling system  1 = no degree^[[3]](#footnote-3)^  2 = secondary school certificate (9 years)^[[4]](#footnote-4)^  3 = secondary school certificate (11 years)^[[5]](#footnote-5)^  4 = advanced technical college certificate^[[6]](#footnote-6)^  5 = high school diploma^[[7]](#footnote-7)^  6 = completed vocational training^[[8]](#footnote-8)^  7 = certified manager^[[9]](#footnote-9)^  8 = certified agriculturist^[[10]](#footnote-10)^  9 = masters certificate in agriculture^[[11]](#footnote-11)^  10 = university degree^[[12]](#footnote-12)^ |
| - 1. An wie vielen Weiterbildungsmaßnahmen (Vorträge, Seminare, Workshops, Beratung generell) nehmen Sie ungefähr pro Jahr teil? | F.8. How many agricultural training workshops do you attend per year on average? | agric training | attendance of agricultural training workshops per year  0 = none  1 = one  2 = two to three  4 = four to five  4 = more than five |
| - 1. Nahmen Sie bereits an dem Beratungsangebot der LWK zum Risikomanagement teil? | F.9. Do you generally attend risk management training provided by extension services? | RM training yes | 1 if yes |
| - - 1. Einmal teilgenommen | F.9.1 Attended once | RM training 1 | 1 if RM training once |
| - - 1. Mehr als einmal teilgenommen | F.9.2 Attended more than once | RM training > 1 | 1 if RM training more than once |

1. Assuming a power utility function U(x)=〖(1-r)〗^(-1) x^(1-r). [↑](#footnote-ref-1)
2. Assuming a power utility function U(x)=〖(1-r)〗^(-1) x^(1-r). [↑](#footnote-ref-2)
3. Schule beendet ohne Abschluss [↑](#footnote-ref-3)
4. Hauptschulabschluss [↑](#footnote-ref-4)
5. Realschulabschluss [↑](#footnote-ref-5)
6. Fachhochschulreife [↑](#footnote-ref-6)
7. Allgemeine Hochschulreife [↑](#footnote-ref-7)
8. Berufsausbildung/Lehre [↑](#footnote-ref-8)
9. Staatlich geprüfter Wirtschafter [↑](#footnote-ref-9)
10. Staatlich geprüfter Agrarbetriebswirt (Landwirt) [↑](#footnote-ref-10)
11. Landwirtschaftsmeister [↑](#footnote-ref-11)
12. Hochschulabschluss (Uni oder FH) [↑](#footnote-ref-12)
