# Supplementary material for: Data on German farmers risk preference, perception and management strategies
Source: Data Brief. 2017 Sep 14;15:102–5. doi: 10.1016/j.dib.2017.09.014 (PMC5614755; doi:10.1016/j.dib.2017.09.014)
Supplement: Supplementary file 5 — Supplementary material [file mmc5.pdf]

Befragung zum landwirtschaftlichen Risikomanagement in NRW

Eine Umfrage der landwirtschaftlichen Fakultät der Universität Bonn

---

Liebe Landwirte, Ihre Meinung ist gefragt!

Was ist Ihre persönliche Einschätzung verschiedener Risikofaktoren, die den landwirtschaftlichen Betrieb betreffen? Und welche Maßnahmen wenden Sie an um das unternehmerische Risiko zu minimieren?

Diesen Fragen gehe ich im Rahmen meiner Promotion an der Universität Bonn nach. Mit Hilfe dieses Fragebogens erhebe ich die dafür notwendigen Informationen aus der Praxis und freue mich über Ihre Unterstützung! Die Ergebnisse werden gerne an Sie zurückgegeben, sodass Sie einen besseren Einblick in Ihr betriebliches Risikoprofil erlangen und somit Ihr Risikomanagement optimieren können. Näheres dazu finden Sie am Ende des Fragebogens.

Bearbeitungszeit: ca. 25 - 30 Minuten

Es werden Fragen zu den folgenden Themenblöcken gestellt:

A. **Risikofaktoren**
B. **Risikoeinstellung**
C. **Umgang mit Wahrscheinlichkeiten**
D. **Betriebliche Informationen**
E. **Risikomanagementstrategien**
F. **Informationen zu Betriebsleitung, Familie und Arbeitskräften**

Die allgemeinen Informationen zu Ihrem Betrieb werden für die statistische Auswertung benötigt. Ich kann Ihnen versichern, dass die Auswertung des Fragebogens unter Einhaltung strengster datenschutzrechtlicher Bestimmungen erfolgt und keine Ihrer Angaben an Dritte weitergegeben werden.

Mit der Teilnahme an dieser Befragung können Sie **bis zu 200€ gewinnen.** Jeder 10te Teilnehmer wird als Gewinner gelost und bekommt einen Betrag basierend auf den Entscheidungen in Teil B ausgezahlt. Um Sie über einen möglichen Gewinn informieren zu können, bitte ich Sie am Ende des Fragebogens auf das gesonderte letzte Blatt Ihre E-Mail Adresse oder Post Adresse zu hinterlassen.

Falls Sie Fragen bezüglich des Fragebogens oder der Auswertung haben, stehe ich Ihnen gerne zur Verfügung!

Bitte schicken Sie den Ausgefüllten Fragebogen in dem beiliegenden portofreien Umschlag bis spätestens zum 31.12.2015 an mich zurück!

Mit freundlichen Grüßen und in gespannter Erwartung auf die Ergebnisse

Manuela Meraner
Wissenschaftliche Mittarbeiterin
Tel: +49 (0) 228 73-2894
Fax: +49 (0)228-73-2758
E-Mail: m.meraner@ilr.uni-bonn.de
Meckenheimer Allee 174
53115 Bonn

A. Risikofaktoren

1. Wie schätzen Sie die Eintrittswahrscheinlichkeit der folgenden Risikoquellen für Ihren Betrieb ein? (von 1 = „sehr unwahrscheinlich“ bis 5 = „sehr wahrscheinlich“)

	sehr unwahr-scheinlich				sehr wahr-scheinlich
	1	2	3	4	5
**Markt- und Preisrisiken**					
Zunehmende Preisschwankungen auf den Absatz-märkten	☐	☐	☐	☐	☐
Zunehmende Preisschwankungen auf den Beschaf-fungsmärkten	☐	☐	☐	☐	☐
Steigende Pachtpreise für Land	☐	☐	☐	☐	☐
Steigende Futtermittelpreise	☐	☐	☐	☐	☐
Schwächung der Erzeuger durch hohe Marktmacht der Abnehmer	☐	☐	☐	☐	☐
**Politikrisiken**					
Weitere Senkungen der EU Direktzahlungen	☐	☐	☐	☐	☐
Verschärfung von Cross Compliance	☐	☐	☐	☐	☐
Steigende Auflagen in der Tierproduktion (Tier-schutz)	☐	☐	☐	☐	☐
Steigende Auflagen in der Pflanzenproduktion (Um-weltschutz)	☐	☐	☐	☐	☐
Weiterer Abbau der EU-Marktstützung (z. Bsp. Au-ßenschutz, Intervention, usw.)	☐	☐	☐	☐	☐
Weitere Ökologisierung der Agrarpolitik	☐	☐	☐	☐	☐
Einschränkungen im landwirtschaftlichen Baurecht	☐	☐	☐	☐	☐
Plötzlicher Wegfall von Absatz- und Bezugsmärkten	☐	☐	☐	☐	☐
**Produktionsrisiken**					
Ertragsschwankungen aufgrund von Klimaänderun-gen	☐	☐	☐	☐	☐
Ertragsausfälle aufgrund von klimatischen Extremer-eignissen (z. Bsp. Hochwasser, Hagel)	☐	☐	☐	☐	☐
Tierseuchen und Tierkrankheiten	☐	☐	☐	☐	☐
Schwierigkeiten in der Bekämpfung von Schadorga-nismen (Resistenzen)	☐	☐	☐	☐	☐
Verringerte Flächenverfügbarkeit	☐	☐	☐	☐	☐

	sehr unwahrscheinlich 1	2	3	4	sehr wahrscheinlich 5
**Finanzielle Risiken**					
Liquiditätsengpässe	☐	☐	☐	☐	☐
Abnahme der Kreditwürdigkeit	☐	☐	☐	☐	☐
**Sonstige Risiken**					
Eingeschränkte Verfügbarkeit qualifizierter Arbeitskräfte	☐	☐	☐	☐	☐
Ausfall von Führungskräften im Betrieb	☐	☐	☐	☐	☐
Probleme bei der Einhaltung von Qualitätsanforderungen	☐	☐	☐	☐	☐
Akzeptanzprobleme der Tierhaltung (z.B. Widerstand gegen Stallneubauten)	☐	☐	☐	☐	☐
Akzeptanzprobleme des Ackerbaus (z.B. Vermaisung der Landschaft)	☐	☐	☐	☐	☐
**Andere Risiken, und zwar:**					
…………………………………………………………………………	☐	☐	☐	☐	☐

2. Wie schätzen Sie die <u>Schadensauswirkung</u> der folgenden Risikoquellen für Ihren Betrieb ein (von 1 = „keine Auswirkungen“ bis 5 = „existenzgefährdend“)?

	keine Auswirkungen				existenzgefährdend
	1	2	3	4	5
**Markt- und Preisrisiken**					
Zunehmende Preisschwankungen auf den Absatzmärkten	☐	☐	☐	☐	☐
Zunehmende Preisschwankungen auf den Beschaffungsmärkten	☐	☐	☐	☐	☐
Steigende Pachtpreise für Land	☐	☐	☐	☐	☐
Steigende Futtermittelpreise	☐	☐	☐	☐	☐
Schwächung der Erzeuger durch hohe Marktmacht der Abnehmer	☐	☐	☐	☐	☐
**Politikrisiken**					
Weitere Senkungen der EU Direktzahlungen	☐	☐	☐	☐	☐
Verschärfung von Cross Compliance	☐	☐	☐	☐	☐
Steigende Auflagen in der Tierproduktion (Tierschutz)	☐	☐	☐	☐	☐
Steigende Auflagen in der Pflanzenproduktion (Umweltschutz)	☐	☐	☐	☐	☐
Weiterer Abbau der EU-Marktstützung (z. Bsp. Außenschutz, Intervention, usw.)	☐	☐	☐	☐	☐
Weitere Ökologisierung der Agrarpolitik	☐	☐	☐	☐	☐
Einschränkungen im landwirtschaftlichen Baurecht	☐	☐	☐	☐	☐
Plötzlicher Wegfall von Absatz- und Bezugsmärkten	☐	☐	☐	☐	☐
**Produktionsrisiken**					
Ertragsschwankungen aufgrund von Klimaänderungen	☐	☐	☐	☐	☐
Ertragsausfälle aufgrund von klimatischen Extremereignissen (z. Bsp. Hochwasser, Hagel)	☐	☐	☐	☐	☐
Tierseuchen und Tierkrankheiten	☐	☐	☐	☐	☐
Schwierigkeiten in der Bekämpfung von Schadorganismen (Resistenzen)	☐	☐	☐	☐	☐
Verringerte Flächenverfügbarkeit	☐	☐	☐	☐	☐

	keine Auswirkungen				existenzgefährdend
	1	2	3	4	5
**Finanzielle Risiken**					
Liquiditätsengpässe	☐	☐	☐	☐	☐
Abnahme der Kreditwürdigkeit	☐	☐	☐	☐	☐
**Sonstige Risiken**					
Eingeschränkte Verfügbarkeit qualifizierter Arbeitskräfte	☐	☐	☐	☐	☐
Ausfall von Führungskräften im Betrieb	☐	☐	☐	☐	☐
Probleme bei der Einhaltung von Qualitätsanforderungen	☐	☐	☐	☐	☐
Akzeptanzprobleme der Tierhaltung (z.B. Widerstand gegen Stallneubauten)	☐	☐	☐	☐	☐
Akzeptanzprobleme des Ackerbaus (z.B. Vermaisung der Landschaft)	☐	☐	☐	☐	☐
**Andere Risiken, und zwar:**					
…………………………………………………………………	☐	☐	☐	☐	☐

B. Risikoeinstellung

Um sicher zu gehen, dass Sie den Auszahlungsmodus zur Belohnung Ihrer Teilnahme verstehen, lesen Sie bitte folgende Erklärung gründlich durch:
Sie sehen unten eine Tabelle mit 10 verschiedenen Szenarien für den Ausgang von zwei Investitionsmöglichkeiten (A und B). Sie entscheiden sich in jedem dieser Szenarien entweder für A oder B. 10 von 100 Teilnehmern werden als Gewinner ausgelost! Wenn Sie dazu gehören, wird Ihr Gewinn folgendermaßen ermittelt:

1) Das Szenario (Zeile), das für die Auszahlung relevant ist, wird zufällig gezogen.
   Zum Beispiel: Wir nehmen an, Szenario 1 wurde gezogen und ihre dort getroffene Entscheidung war Investitionsmöglichkeit A.
2) Anschließend wird das Ergebnis des Szenarios anhand der dort vorgegebenen Wahrscheinlichkeitsverteilung zufällig gezogen.
   Zum Beispiel: In Szenario 1 wird dann aus einem Topf aus 10 Kugeln (eine grüne und neun blaue), zufällig Ihre Auszahlung gezogen (80.000 € oder 100.000 €). Wird eine blaue Kugel gezogen, ergibt sich eine Auszahlung von 80.000 €. Dieser Betrag wird durch 1.000 geteilt und an Sie ausgezahlt. Ihnen werden somit 80€ Gewinn übermittelt!

3. Nehmen Sie an, Ihnen wird angeboten eine landwirtschaftliche Investition zu tätigen. Dabei erhalten Sie mit bestimmten Wahrscheinlichkeiten für Investition A eine Auszahlung von 100.000 € oder 80.000 € und für Investition B eine Auszahlung von 192.500 € oder 5.000 €. Beide Investitionen unterscheiden sich nicht bezüglich der Kosten und Auszahlungszeitpunkte. Sie können in der folgenden Tabelle in jeder Zeile zwischen den zwei Investitionsentscheidungen (A oder B) wählen.

	A		B		A	B
**1**	10% Wahrscheinlichkeit für eine Auszahlung von 100.000 € und 90% Wahrscheinlichkeit für eine Auszahlung von 80.000 €	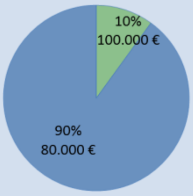	10% Wahrscheinlichkeit für eine Auszahlung von 192.500 € und 90% Wahrscheinlichkeit für eine Auszahlung von 5.000 €	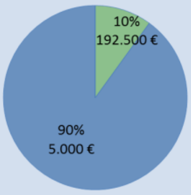	☐	☐
**2**	20% Wahrscheinlichkeit für eine Auszahlung von 100.000 € und 80% Wahrscheinlichkeit für eine Auszahlung von 80.000 €	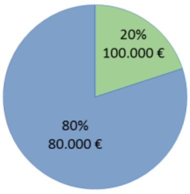	20% Wahrscheinlichkeit für eine Auszahlung von 192.500 € und 80% Wahrscheinlichkeit für eine Auszahlung von 5.000 €	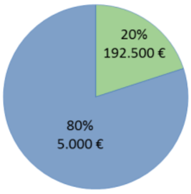	☐	☐
**3**	30% Wahrscheinlichkeit für eine Auszahlung von 100.000 € und 70% Wahrscheinlichkeit für eine Auszahlung von 80.000 €	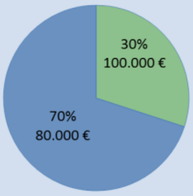	30% Wahrscheinlichkeit für eine Auszahlung von 192.500 € und 70% Wahrscheinlichkeit für eine Auszahlung von 5.000 €	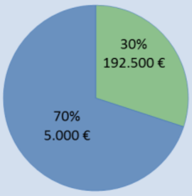	☐	☐
4	40% Wahrscheinlichkeit für eine Auszahlung von 100.000 € und 60% Wahrscheinlichkeit für eine Auszahlung von 80.000 €	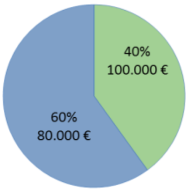	40% Wahrscheinlichkeit für eine Auszahlung von 192.500 € und 60% Wahrscheinlichkeit für eine Auszahlung von 5.000 €	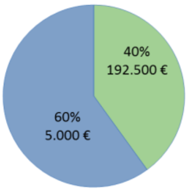	☐	☐

	A	B	A	B
5	50% Wahrscheinlichkeit für eine Auszahlung von 100.000 € und 50% Wahrscheinlichkeit für eine Auszahlung von 80.000 € 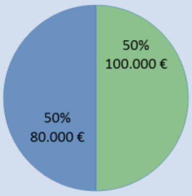	50% Wahrscheinlichkeit für eine Auszahlung von 192.500 € und 50% Wahrscheinlichkeit für eine Auszahlung von 5.000 € 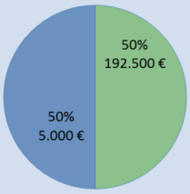	☐	☐
6	60% Wahrscheinlichkeit für eine Auszahlung von 100.000 € und 40% Wahrscheinlichkeit für eine Auszahlung von 80.000 € 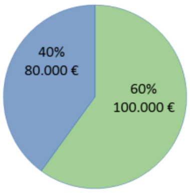	60% Wahrscheinlichkeit für eine Auszahlung von 192.500 € und 40% Wahrscheinlichkeit für eine Auszahlung von 5.000 € 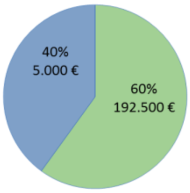	☐	☐
7	70% Wahrscheinlichkeit für eine Auszahlung von 100.000 € und 30% Wahrscheinlichkeit für eine Auszahlung von 80.000 € 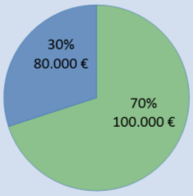	70% Wahrscheinlichkeit für eine Auszahlung von 192.500 € und 30% Wahrscheinlichkeit für eine Auszahlung von 5.000 € 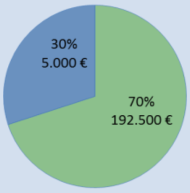	☐	☐
8	80% Wahrscheinlichkeit für eine Auszahlung von 100.000 € und 20% Wahrscheinlichkeit für eine Auszahlung von 80.000 € 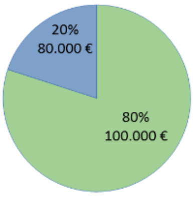	80% Wahrscheinlichkeit für eine Auszahlung von 192.500 € und 20% Wahrscheinlichkeit für eine Auszahlung von 5.000 € 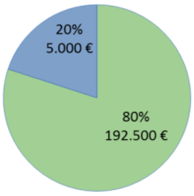	☐	☐
9	90% Wahrscheinlichkeit für eine Auszahlung von 100.000 € und 10% Wahrscheinlichkeit für eine Auszahlung von 80.000 € 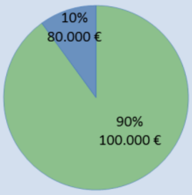	90% Wahrscheinlichkeit für eine Auszahlung von 192.500 € und 10% Wahrscheinlichkeit für eine Auszahlung von 5.000 € 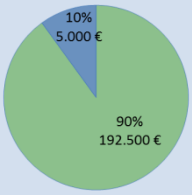	☐	☐
10	100% Wahrscheinlichkeit für eine Auszahlung von 100.000 € und 0% Wahrscheinlichkeit für eine Auszahlung von 80.000 € 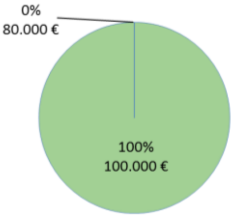	100% Wahrscheinlichkeit für eine Auszahlung von 192.500 € und 0% Wahrscheinlichkeit für eine Auszahlung von 5.000 € 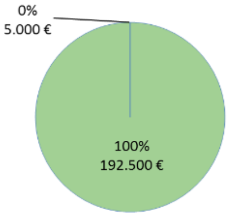	☐	☐

Bitte entscheiden Sie sich **in jeder Zeile** für eine Investition in A oder B.

4. Wie schätzen Sie sich persönlich ein: Sind Sie im Allgemeinen ein risikobereiter Mensch oder versuchen Sie, Risiken zu vermeiden (von 0 = „gar nicht risikobereit“ bis 10 = „sehr risikobereit“).

**gar nicht risikobereit**										**sehr risikobereit**
0	1	2	3	4	5	6	7	8	9	10
☐	☐	☐	☐	☐	☐	☐	☐	☐	☐	☐

5. **Wie zufrieden sind Sie gegenwärtig, alles in allem, mit Ihrem Leben? (von 1 = „unzufrieden“ bis 10 = „zufrieden“)**

unzufrieden 1	2	3	4	5	6	7	8	9	zufrieden 10
☐	☐	☐	☐	☐	☐	☐	☐	☐	☐

6. **Und was glauben Sie, wie wird es wohl in einem Jahr sein? (von 1 = „unzufrieden“ bis 10 = „zufrieden“)**

unzufrieden 1	2	3	4	5	6	7	8	9	zufrieden 10
☐	☐	☐	☐	☐	☐	☐	☐	☐	☐

7. **Bitte geben Sie in der folgenden Tabelle an inwiefern Sie den Aussagen zustimmen (von 1 = „stimme voll zu“ bis 5 = „lehne ab“).**

	stimme voll zu				lehne ab
	1	2	3	4	5
Ich bin bereit, in Bezug auf die Produktion mehr Risiken einzugehen als andere Landwirte.	☐	☐	☐	☐	☐
Ich bin bereit, in Bezug auf Markt- und Preise mehr Risiken einzugehen als andere Landwirte.	☐	☐	☐	☐	☐
Ich bin bereit, in Bezug auf Fremdkapitalaufnahme mehr Risiken einzugehen als andere Landwirte.	☐	☐	☐	☐	☐
Ich bin bereit, in Bezug auf Landwirtschaft generell mehr Risiken einzugehen als andere Landwirte.	☐	☐	☐	☐	☐

8. **Durch welchen der folgenden Faktoren hatten Sie in den letzten 5 Jahren größere Verluste auf Ihrem landwirtschaftlichen Betrieb (mehrfach Nennung möglich)?**

☐ Markt- und Preisrisiken

☐ Politikänderungen

☐ Produktionsrisiken

☐ Finanzielle Risiken

☐ Risiken durch Arbeitskräfte, gesellschaftliche Akzeptanz

☐ keine größeren Verluste in den letzten 5 Jahren

C. Umgang mit Wahrscheinlichkeiten

9. Wie schätzen Sie sich im Bruchrechnen ein?

sehr gut					überhaupt nicht gut
1	2	3	4	5	6
☐	☐	☐	☐	☐	☐

10. Wie schätzen Sie Ihre Fähigkeiten im Umgang mit Prozentwerten ein?

sehr gut					überhaupt nicht gut
1	2	3	4	5	6
☐	☐	☐	☐	☐	☐

11. Wie gut sind Sie darin einzuschätzen, wie viel ein Ferkel bei einer 25%-gen Preisreduzierung kostet?

sehr gut					überhaupt nicht gut
1	2	3	4	5	6
☐	☐	☐	☐	☐	☐

12. Wenn Sie eine Tageszeitung lesen, wie nützlich finden Sie Tabellen und Diagramme als Teil eines Artikels?

sehr nützlich					überhaupt nicht nützlich
1	2	3	4	5	6
☐	☐	☐	☐	☐	☐

13. Wenn Ihnen jemand etwas über die Wahrscheinlichkeit erzählt, dass ein bestimmtes Ereignis eintreffen wird, bevorzugen Sie es dann, wenn dazu Worte benutzt werden („passiert selten“) oder wenn Zahlenwerte benutzt werden („es gibt eine 1%ige Wahrscheinlichkeit“)

bevorzuge immer Worte					bevorzuge immer Prozentwerte
1	2	3	4	5	6
☐	☐	☐	☐	☐	☐

14. Wenn Sie einen Wetterbericht hören, bevorzugen Sie es dann, wenn die Vorhersagen in Prozentwerten ausgedrückt werden (z.B. „es gibt heute eine 20%ige Regenwahrscheinlichkeit“) oder in Worten(z.B. „heute ist die Regenwahrscheinlichkeit gering“)?

bevorzuge immer Prozentwerte					bevorzuge immer Worte
1	2	3	4	5	6
☐	☐	☐	☐	☐	☐

15. Wie oft finden Sie Informationen, die in Zahlen ausgedrückt sind, nützlich?

Sehr oft					nie
1	2	3	4	5	6
☐	☐	☐	☐	☐	☐

D. Betriebliche Informationen

16. Wird Ihr landwirtschaftlicher Betrieb im Haupt- oder im Nebenerwerb geführt?

☐ Haupterwerb ☐ Nebenerwerb

17. Wird Ihr landwirtschaftlicher Betrieb konventionell oder ökologisch bewirtschaftet?

☐ Konventionell ☐ Ökologisch

18. Wie ist die Flächenausstattung (in ha) in Ihrem Betrieb organisiert?

	ha
Landw. genutzte Fläche	
- davon Eigentum	
Ackerfläche	
- davon Sonderkulturen	
Grünland	

19. Welchen Tierbestand gibt es in Ihrem Betrieb?

	Bestand
Zuchtsauen	
Mastschweine (ab 25kg)	
Milchvieh	
Mastbullen	
Mastgeflügel	
Legehennen	
Sonstiges: ……………………………………….	

20. Ist auf Ihrem Betrieb die Hofnachfolge gesichert?

☐ ja ☐ eher schon ☐ eher nicht ☐ nein

☐ nein, es steht keine Hofübernahme in den nächsten 15 Jahren an
☐ nein, auslaufender Betrieb

E. Risikomanagementstrategien

21. Welche der folgenden Risikomanagementstrategien setzen Sie auf Ihrem Betrieb ein (mehrfach Angaben möglich)?

Risikoangepasste Produktionsweise (z. B. Vorsichtige Wahl der Aussaatzeitpunkte)	☐
Nutzung resistenter Sorten oder robuster Rassen	☐
Landwirtschaftliche Diversifizierung (z. B. Kombinationen von Winter uns Sommergetreiden, Tierhaltung und Pflanzenanbau)	☐
Landwirtschaftsnahe Diversifizierung (z. B. Direktvermarktung, Fremdenverkehr, Hof Café, Energieerzeugung, Sonstiges)	☐
Investitionen in Technologien, die meine Produktionsbedingungen besser an das Wetter anpassen (z. B. Bewässerung)	☐
Bildung von Liquiditätsreserven	☐
Arbeiten außerhalb des Betriebs	☐
Investitionen außerhalb des Betriebs (z.B. andere Unternehmungen, Immobilien, Wertanlagen)	☐
Versicherungen:	
Ertragsschadenversicherung (z.B. Tierversicherung)	☐
Hagelversicherung	☐
Mehrgefahrenversicherung (z.B. Hagel + Sturm und Starkregen, Hagel + Sturm und Starkregen + Starkfrost)	☐
Betriebshaftpflichtversicherung	☐
Betriebshaftpflichtversicherung + Bodenkasko, erweiterte Umwelthaftpflicht	☐
Rechtschutzversicherung	☐
Warenterminbörsen	☐
Härter Arbeiten /private Ausgaben kürzen	☐
Kooperationen mit anderen Betrieben	☐
Keine dieser Maßnahmen	☐
Sonstige, und zwar	....

universitätbonn landwirtschaftliche fakultät

F. Betriebsleitung, Familie und Arbeitskräfte

22. Wie viele Personen einschließlich Ihnen leben in Ihrem Haushalt (inkl. Altenteiler)?

[ ] Personen

23. In welchem Jahr sind Sie geboren?

| 1 | 9 | | |

24. Bitte geben Sie Ihr Geschlecht an:

☐ männlich ☐ weiblich

25. Wie viele Arbeitskräfte arbeiten insgesamt Vollzeit auf Ihrem landwirtschaftlichen Betrieb (inkl. Familienarbeitskräfte)?

[ ] Personen

26. In welchem Jahr haben Sie den Betrieb übernommen?

| | | | |

27. Bitte geben Sie Ihre Postleitzahl an:

| | | | | |

28. Bitte geben Sie Ihren <u>höchsten</u> Bildungsabschluss an.

☐ Schule beendet ohne Abschluss
☐ Hauptschulabschluss
☐ Realschulabschluss
☐ Fachhochschulreife
☐ Allgemeine Hochschulreife

☐ Berufsausbildung/Lehre
☐ Staatlich geprüfter Wirtschafter
☐ Staatlich geprüfter Agrarbetriebswirt (Landwirt)
☐ Landwirtschaftsmeister
☐ Hochschulabschluss (Uni oder FH)
☐ anderer Schulabschluss ……………………….

29. An wie vielen Weiterbildungsmaßnahmen (Vorträge, Seminare, Workshops, Beratung generell) nehmen Sie ungefähr pro Jahr teil?

☐ keine ☐ 1 ☐ 2 - 3 ☐ 4 - 5 ☐ mehr als 5

30. Nahmen Sie bereits an dem Beratungsangebot der LWK zum Risikomanagement?

☐ ja wie häufig? ☐ 1 mal ☐ mehr als 1 mal
☐ nein

31. Haben Sie abschließend noch Anmerkungen?

………
………
………

Sie sind am Ende unserer Umfrage angelangt.

Herzlichen Dank für Ihre Unterstützung!

Bitte schicken Sie den Ausgefüllten Fragebogen in dem beiliegenden portofreien Umschlag bis spätestens zum 31.12.2015 an mich zurück

Mit freundlichen Grüßen Manuela Meraner

Um Ihnen den möglichen Gewinn zu übermitteln, tragen Sie bitte Ihre Adresse oder E-Mail Adresse ein.

Möchten Sie die Auswertungen der Umfrage als Bericht zugesandt bekommen?

☐ Ja, bitte senden Sie mir einen Bericht Ihrer Auswertungen zu. ☐ Nein

Adresse: ....

oder

E-Mail: ....
